# Supplementary material for: Nitrous oxide activates layer 5 prefrontal neurons via SK2 channel inhibition for antidepressant effect
Source: Nat Commun. 2025 Apr 3;16:2999. doi: 10.1038/s41467-025-57951-y (PMC11968965; doi:10.1038/s41467-025-57951-y)
Supplement: Supplementary file 1 — Supplementary Information [file 41467_2025_57951_MOESM1_ESM.pdf]

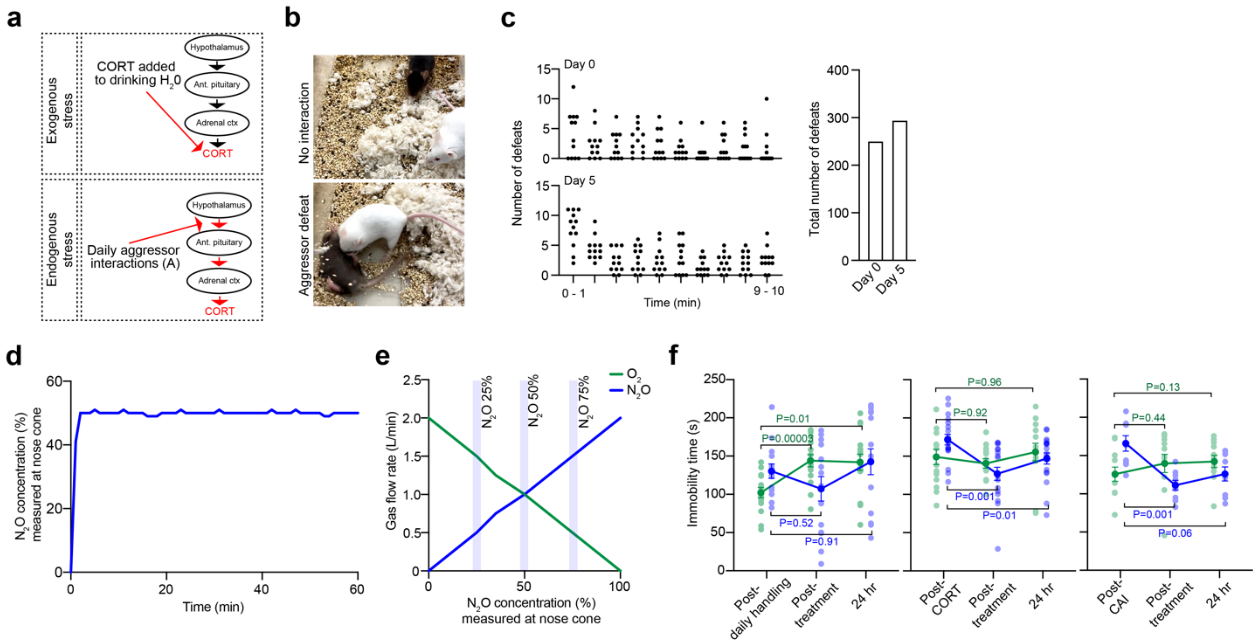

Supplemental Fig. 1. N_2O exposure induces a sustained antidepressant-like response in chronically stressed mice but not wild-type mice.

a, Left, schematic showing two different methods for driving the hypothalamus-pituitary-adrenal axis. Corticosterone added to drinking water for 21 days represents the exogenous manipulation (top) whereas male C57/BL6 mice exposed to daily interactions (10 min/day) with a male CD-1 (screened for aggression) mouse for 10 days is referred to as the endogenous manipulation (bottom).

b, Representative defeat experienced by C57BL/6 mouse exposed to screened aggressor mouse. C57BL/6 mouse is often forced on its backside (lower image).

c, Left, number of defeats over a 10 min period on first day of pairing (day 0) and day 5 experienced by 12 C57BL/6 mice. Right, total number of defeats experienced by same cohort on day 0 and day 5.

d, N_2O concentration sampled by Philips clinical gas monitoring system at nose cone during two-photon recording.

e, Blended gas mixtures for different N_2O concentrations, i.e. 25%, 50%, and 75%, while total output remains at 2 liters/min.

f, Serial tail suspension measurements were performed in control (left; O_2, *n* = 15; N_2O, *n* = 7), CORT (middle; O_2, *n* = 13; N_2O, *n* = 18), and CAI (right; O_2, *n* = 11; N_2O, *n* = 9) mice before and after oxygen (O_2) or N_2O exposure at 1 and 24 hr. Chronically stressed mice exposed to N_2O, but not O_2, for 1 hr developed a sustained decrease in immobility at 1 hr post treatment that persisted up to 24 hr (two-way ANOVA time x treatment: control, $F_{(1.4, 38)}$ = 4.1, P = 0.004; CORT, $F_{(1.8, 53)}$ = 5.8, P = 0.03; CAI, $F_{(1.8, 33)}$ = 4.6, P = 0.00002 with post hoc Sidak's multiple comparisons shown in each panel.

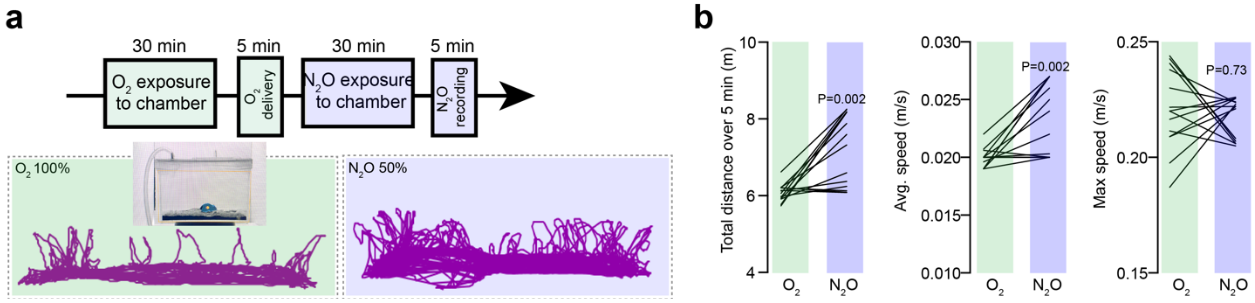

Supplemental Fig. 2. N_2O exposure at 50% increases animal movement and exploration.
a, The effect of O_2 (100%, top) or N_2O (50%, bottom) on animal's spontaneous movement (not head-fixed; n = 15). A representative 5 min tracing (produced by AnyMaze) of an individual animal's movement across closed chamber after 30 min of gas exposure (to ensure equilibrium). Horizontal lines capture movement across chamber whereas vertical lines note exploratory movements. N_2O increased both the horizontal and vertical movements.
b, Total distance, average speed, and max speed over 5 min period of all mice tested in O_2 and then N_2O. N_2O significantly increased the total distance (Wilcoxon matched-pairs signed rank test, $P = 0.002$) and average speed (Wilcoxon matched-pairs signed rank test, $P = 0.002$), but not max speed (Wilcoxon matched-pairs signed rank test, $P = 0.73$).

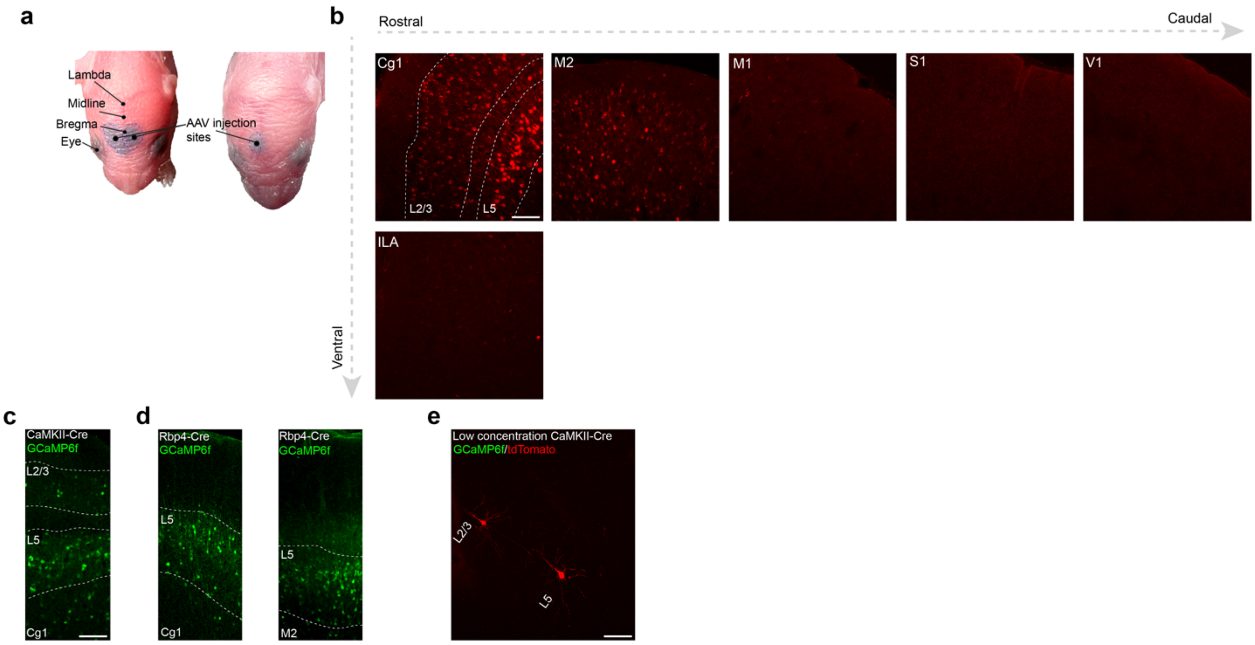

Supplemental Fig. 3. Postnatal adeno-associated virus injection strategy enables genetically encoded sensor targeting to prefrontal cortical regions.

a, Photograph of postnatal day 1 pup injected with cocktail of AAV and dye in a bilateral (left) or unilateral (right) approach. One postnatal day 1-2, landmarks (hemispheres, lambda, bregma, eyes, etc.) can be visible which aids in successful injection and AAV targeting.

b, Postnatal injection of AAVs harboring CaMKII-Cre and Cre-dependent tdTomato into medial prefrontal cortex (PFC). Coronal sections demonstrate the expression of tdTomato in layer 2/3 (L2/3) and layer 5 (L5) in Cg1 and M2 of PFC but not in neighboring areas (primary motor cortex (M1) or infralimbic cortex (ILA)) or in distant regions (primary somatosensory (S1) or visual cortices (V1)).

c, Coronal sections depicting AAV encoding CaMKII-Cre drove GCaMP6f expression in L2/3 and L5 of Cg1.

d, Transgenic Rbp4-Cre mice drove specific layer 5 in Cg1 (left) or M2 regions (right).

e, Diluted AAV-CaMKII-Cre produced sparse labelling of L2/3 and/or L5 neurons. Sparse labelling is critical to structure-function mapping of individual L5 neurons (Fig. 4). Scale bar, 100 μm for all images.

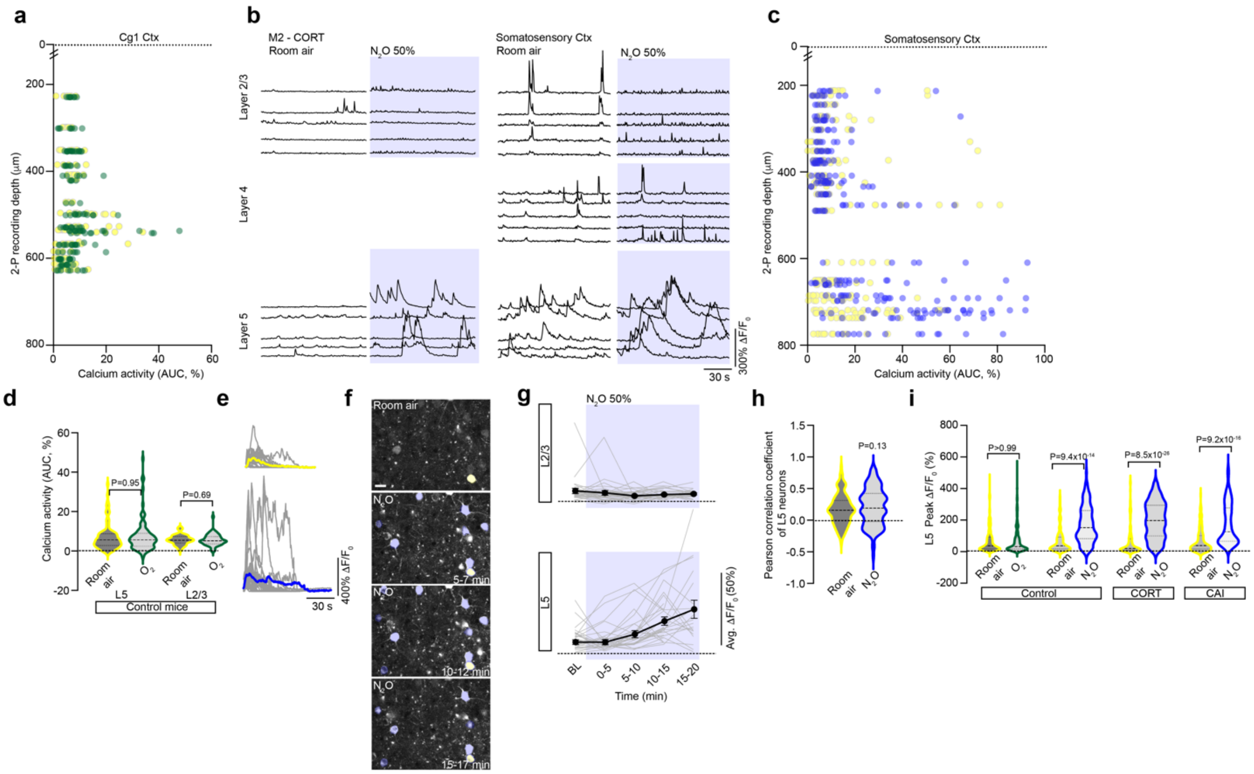

Supplemental Fig. 4. N_2O, but not oxygen therapy, recruits Layer 5 neuronal activity.

a, All neurons recorded in layer 2/3 (L2/3) or layer 5 (L5) under room air and O_2 therapy (100%) from across the Cg1 cortical column. Note the overlapping activity of excitatory neurons between wakefulness/room air (yellow) and O_2 (green).

b, Left, representative GCaMP6 traces of individual pyramidal neurons from L2/3 or L5 of M2 region under room air and N_2O inhalation in CORT-treated mouse. Right, representative GCaMP6 traces of individual pyramidal neurons spanning from L2/3 to L5 in the somatosensory cortex (S1) under room air and N_2O.

c, All neurons recorded from across the S1 cortical column. Note the N_2O-induced activation of L5 neurons a depth of >600 μm.

d, Average calcium response of L5 (n = 96) and L2/3 neurons (n = 56) under wakefulness/room air and O_2 therapy in control mice. O_2 fails to activate either cell type (Wilcoxon matched-pairs signed rank: L5, P = 0.95, L2/3, P = 0.69).

e, Individual transients from L5 neuron under room air (top) and N_2O (bottom). Note the sustained calcium elevation under N_2O condition.

f, Two-photon movies (2 min time-series) of L5 neurons collapsed into a single image over various time points into a N_2O inhalational event. L5 neurons are repetitively activated under N_2O (shaded blue). Scale bar, 20 μm.

g, Average (black line) calcium activity of L2/3 (top; n = 33 cells from 3 mice) and L5 (bottom; n = 30 cells from 3 mice) pyramidal neurons (individual cells shown with gray lines) before and after starting N_2O inhalation (50%).

h, Pearson correlation coefficient measurements between pairs of L5 neurons under room air and N_2O (n = 122; Wilcoxon matched-pairs signed rank, P = 0.13).

i, Peak $\Delta F/F_0$ of L5 neurons under room air (yellow) and N_2O (blue) in control and chronically stressed mice. N_2O induced a robust increase in peak signals across all conditions (Kruskal-Wallis (312): $P = 1.1 \times 10^{-63}$ followed by Dunn's multiple comparisons, WT O_2: $P > 0.99$; WT N_2O: $P = 9.4 \times 10^{-14}$; CORT N_2O: $P = 8.5 \times 10^{-26}$; CAI N_2O: $P = 9.2 \times 10^{-16}$).

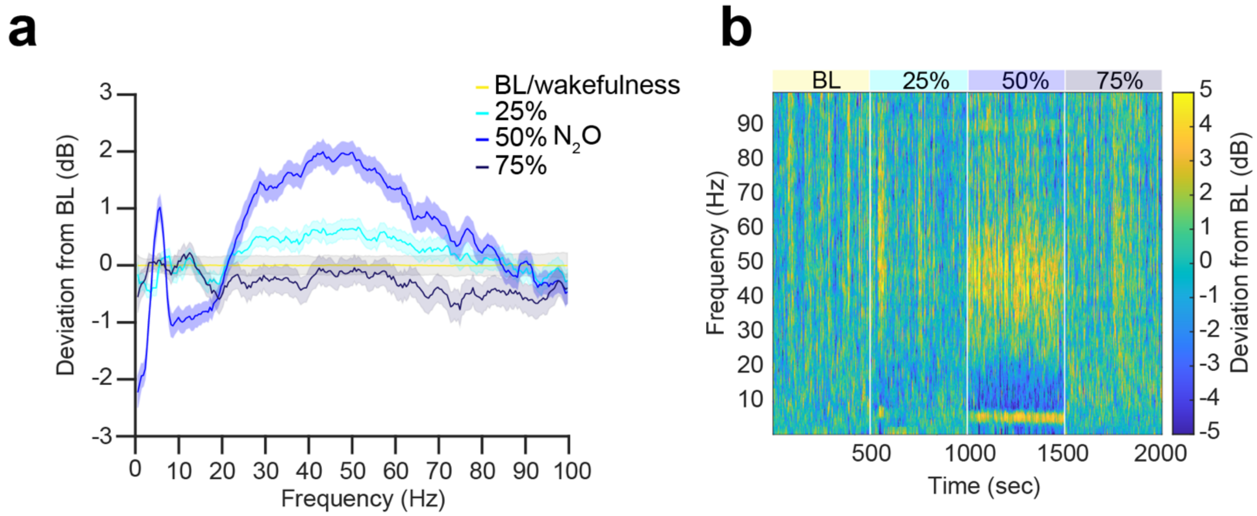

Supplemental Fig. 5. N_2O at 50% induces an activated electroencephalogram.

a, Differences in power across EEG frequencies induced by N_2O (25, 50, 75%) from its baseline measures recorded under room air conditions.

b, Power spectrogram across increasing N_2O concentrations. Note the increase in higher frequency oscillations with inhaled N_2O 50%.

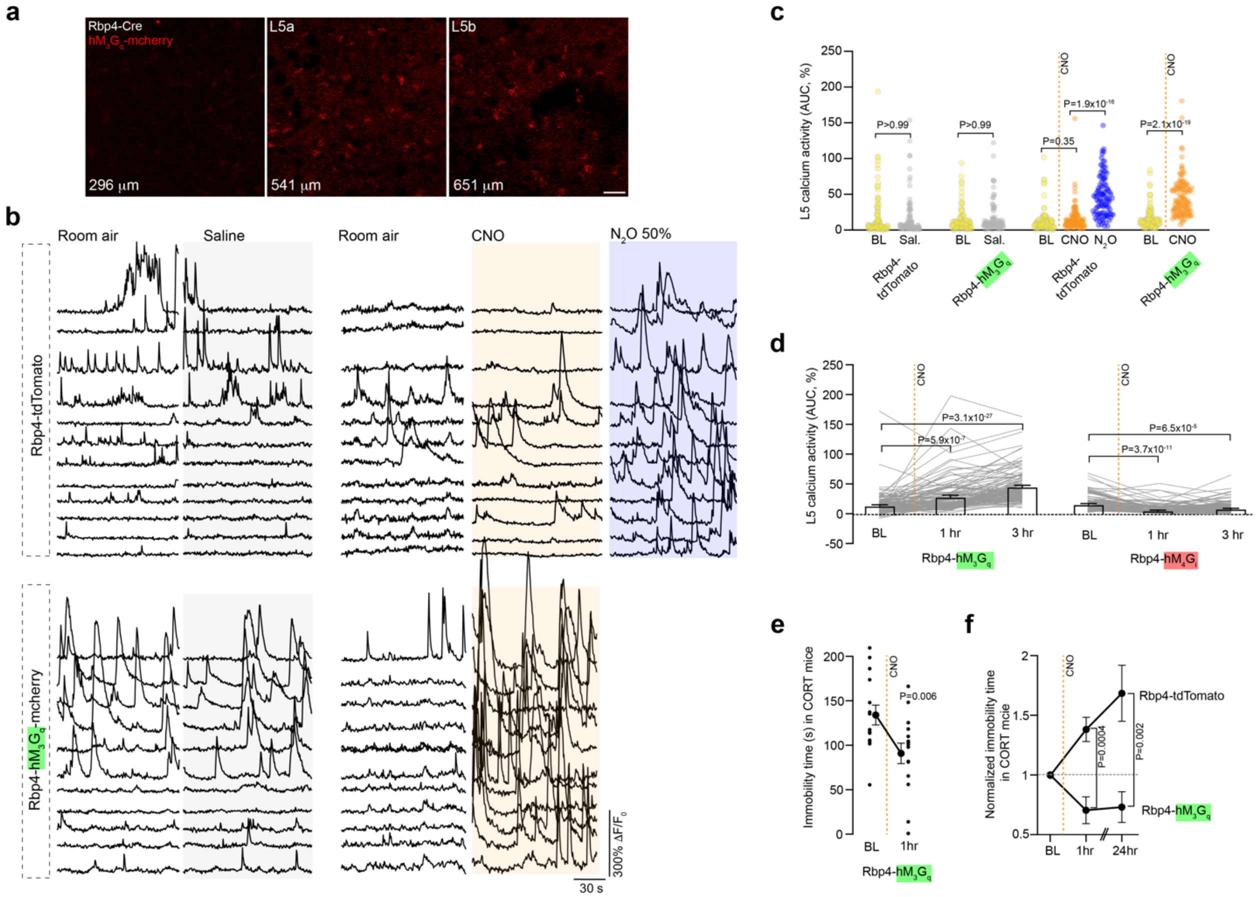

Supplemental Fig. 6. L5 neuronal expression of DREADD-hM$_3$G$_q$-mcherry and its activation drives persistent neuronal activity.

a, Two-photon images of hM$_3$G$_q$-mcherry expression in Rbp4-Cre mice. hM$_3$G$_q$-mcherry was expressed in L5a and L5b (right two images) but not layer 2/3 (left image). Scale bar, 20 µm.

b, Representative GCaMP6 traces of individual L5 neurons from Rbp4 mice expressing either tdTomato (top) or DREADD hM$_3$G$_q$-mcherry (bottom) under saline (tdTomato: n = 100 cells from 3 mice; hM$_3$G$_q$: n = 99 cells from 3 mice) or CNO conditions (tdTomato: n = 106 cells from 3 mice; hM$_3$G$_q$: n = 125 cells from 3 mice).

c, Summary of cellular responses from **b**. No significant effects ((Kruskal-Wallis (328): $P < 1.0 \times 10^{-15}$ followed by Dunn's multiple comparisons, P>0.35) on L5 activity were observed in mice expressing DREADD that received saline, or in tdTomato-expressing mice that received CNO. Strong increases in L5 activity were only seen in DREADD-expressing mice upon CNO administration ($P = 2.1 \times 10^{-19}$). Moreover, we observed that N_2O robustly activated L5 neurons even in the presence of CNO (n = 106 cells from 3 mice; $P = 1.9 \times 10^{-16}$).

d, Average calcium response of L5 neurons expressing either hM$_3$G$_q$-mcherry (left; n = 108 cells from 3 mice) or hM$_4$G$_i$-mcherry (right) before and after CNO injection. CNO-induced activation of L5-hM$_3$G$_q$ drove an increased activity state over hours (Kruskal-Wallis (118): $P = 2.0 \times 10^{-27}$ followed by Dunn's multiple comparisons, 1 hr: $P = 5.9 \times 10^{-7}$; 3 hr: $P = 3.1 \times 10^{-27}$). In contrast, L5-hM$_4$G$_i$ (right; n = 90 cells from 3 mice) activation induced a persistent hypoactivity over hours (Kruskal-Wallis (46): $P = 1.1 \times 10^{-10}$ followed by Dunn's multiple comparisons, 1hr: $P = 3.7 \times 10^{-11}$; 3hr: $P = 6.5 \times 10^{-5}$).

e, Immobility time during tail suspension in CORT treated Rbp4-Cre mice (n = 15) expressing hM$_3$G$_q$ bilaterally in PFC before and after CNO injection (Paired t-test (t = 3.3, df =14): P = 0.006).

f, Sustained decrease in immobility time during TST following a single injection of CNO in CORT treated Rbp4-Cre mice expressing hM3Gq bilaterally in PFC (n = 7) as opposed to Rbp4-

Cre mice expressing tdTomato (n = 11; Mann Whitney rank sum test: 1 hr, P = 0.0004; 24 hr, P = 0.002).

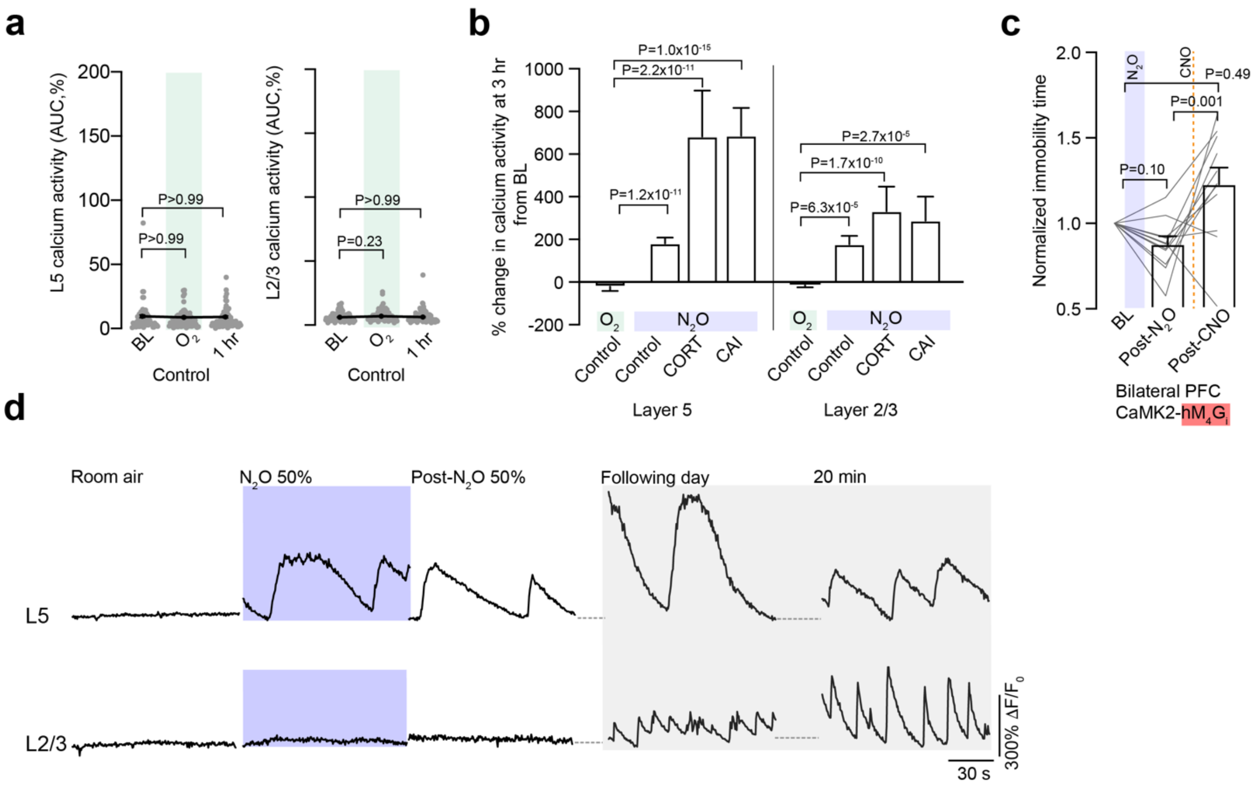

Supplemental Fig. 7. N_2O drives persistent L5 neuronal activity following exhalation.

a, Average calcium responses of L5 and L2/3 neurons under room air, O_2, and following O_2 therapy at 1 hr. O_2 failed to recruit either cell types following gas removal (WT L5 O_2, *n* = 74 cells from 3 mice, Kruskal-Wallis (0.07): *P* = 0.96; WT L2/3, *n* = 99 cells from 3 mice, Kruskal-Wallis (3): *P* = 0.19). Dunn's multiple comparisons shown in plot.

b, Percentage change in spontaneous activity at 3 hr time point from N_2O exposure normalized to its baseline recorded under room air conditions. At 3 hr timepoint, control (*n* = 133 cells from 3 mice), CORT (*n* = 134 cells from 3 mice) and CAI (*n* = 174 cells from 3 mice) mice show persistent L5 (Kruskal-Wallis (147.6): $P < 1.0 \times 10^{-15}$ followed by Dunn's multiple comparisons, control, $P = 1.2 \times 10^{-11}$; CORT, $P = 2.2 \times 10^{-11}$; CAI, $P = 1.0 \times 10^{-15}$) and L2/3 (control, $P = 6.3 \times 10^{-5}$; CORT, $P = 1.7 \times 10^{-10}$; CAI, $P = 2.7 \times 10^{-5}$) activity as compared to O_2 treated control mice (*n* = 192 cells from 3 mice).

c, Immobility time of mice expressing CaMK2-hM_4G_i in PFC bilaterally (*n* = 10 mice) across N_2O exposure followed by CNO-induced inhibition of both L2/3 and L5 activity in this region. CNO-induced hM_4G_i activation promoted the reversal N_2O-induced behavioral effect (Kruskal-Wallis (13): *P* = 0.002 followed by Dunn's multiple comparisons, BL vs. Post N_2O: *P* = 0.10; Post N_2O vs. Post CNO: *P* = 0.001; BL vs. Post CNO: *P* = 0.49).

d, Representative traces from L5 and L2/3 cells that become activated during N_2O and at multiple timepoints following the N_2O exposure.

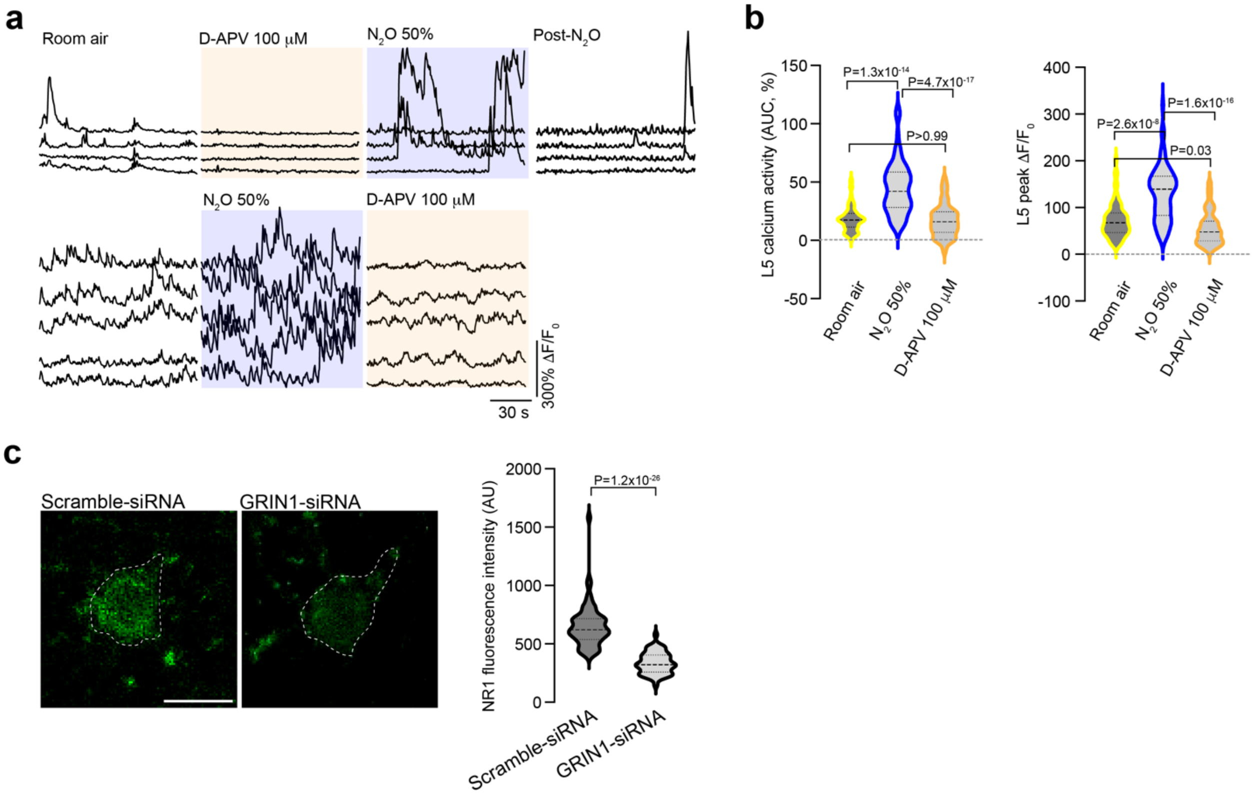

Supplemental Fig. 8. Persistent L5 neuronal activity induced by N_2O reduced by NMDA-receptor inhibition follow exhalation.

a, Top, representative GCaMP6 traces from L5 responses following local application of D-APV (100 μM), N_2O, 1hr after N_2O. N_2O robustly activated these L5 neurons but persistent activity post-N_2O was dampened. Bottom, traces of individual L5 neurons exposed to N_2O and then D-APV. Post-N_2O L5 responses were suppressed in the presence of D-APV.

b, Average calcium activity and peak responses of L5 neurons (n = 81 cells from 3 mice) under room air, N_2O, followed by D-APV. D-APV significantly reduced N_2O-induced responses to baseline measurements (Avg. calcium, Kruskal-Wallis (90): $P = 3.2 \times 10^{-20}$ followed by Dunn's multiple comparisons, $P > 0.99$; Peak calcium, Kruskal-Wallis (73): $P = 1.1 \times 10^{-16}$ followed by Dunn's multiple comparisons, $P = 0.03$).

c, Representative two-photon images (left) and quantification (right) of NR1 fluorescence intensity in cultured cortical neurons infected with either scramble-siRNA (n = 65 cells) or GRIN1-siRNA (n = 56 cells) immunostained against NR1. GRIN1-siRNA significantly reduced NR1 signals (Mann-Whitney rank sum test, $P = 1.2 \times 10^{-26}$). Scale bar, 20 μm.

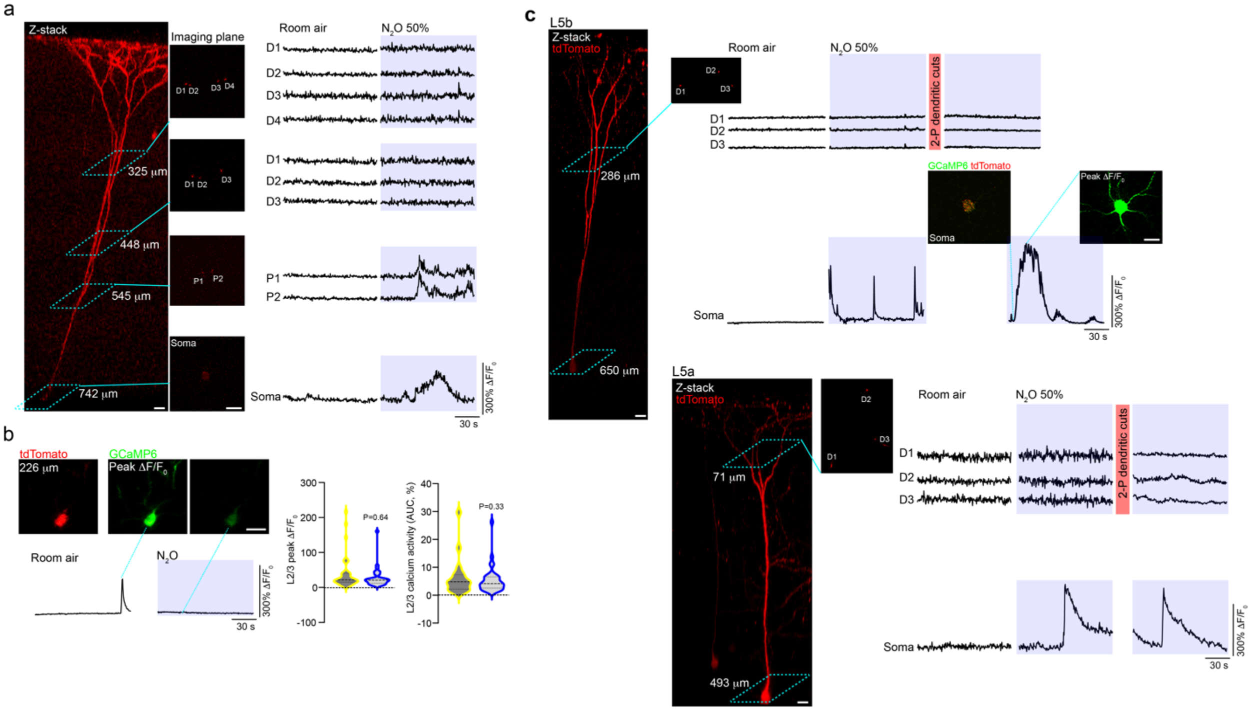

Supplemental Fig. 9. Sparse labelling of individual L5 neurons with GCaMP6 and tdTomato enables two-photon imaging across dendritic compartments to soma.

a, Left, two-photon Z-stack of an individual L5 neuron imaged at several different locations (teal boxes connected to 2-D imaging plane). Right, GCaMP6 traces corresponding to labelled ROIs. N_2O (blue shaded region) recruited L5 soma and deep parent (P) dendrite whereas superficial dendrites (D) remained inactive during the treatment.

b, Left, two-photon images (top) and GCaMP6 traces (bottom) of an individual L2/3 neuron labelled with GCaMP6 and tdTomato. Right, peak and average calcium responses of all individual L2/3 cells recorded (n = 35 from 11 mice) recorded under sparse labelling conditions (dilate CaMK2-Cre; cells/mice different from those reported in Fig. 1 and 2). N_2O inhalation failed to activate these cells over their activity defined in wakefulness/room air (Wilcoxon matched-pairs signed rank: peak calcium, P = 0.64; avg. calcium, P = 0.33).

c, Left, two-photon Z-stack of L5 neuron (top - L5b, bottom - L5a) with two imaging planes marked, one dendritic location with 3 dendritic ROIs and one deep region corresponding to soma. 2 L5 neurons are from 2 different mice. Right, GCaMP6 traces of dendritic ROIs and soma show inactive baseline. N_2O activated the soma without corresponding recruitment of dendrites. Two-photon laser cuts to dendritic ROIs did not change spontaneous somatic responses. Scale bars, 20 μm.

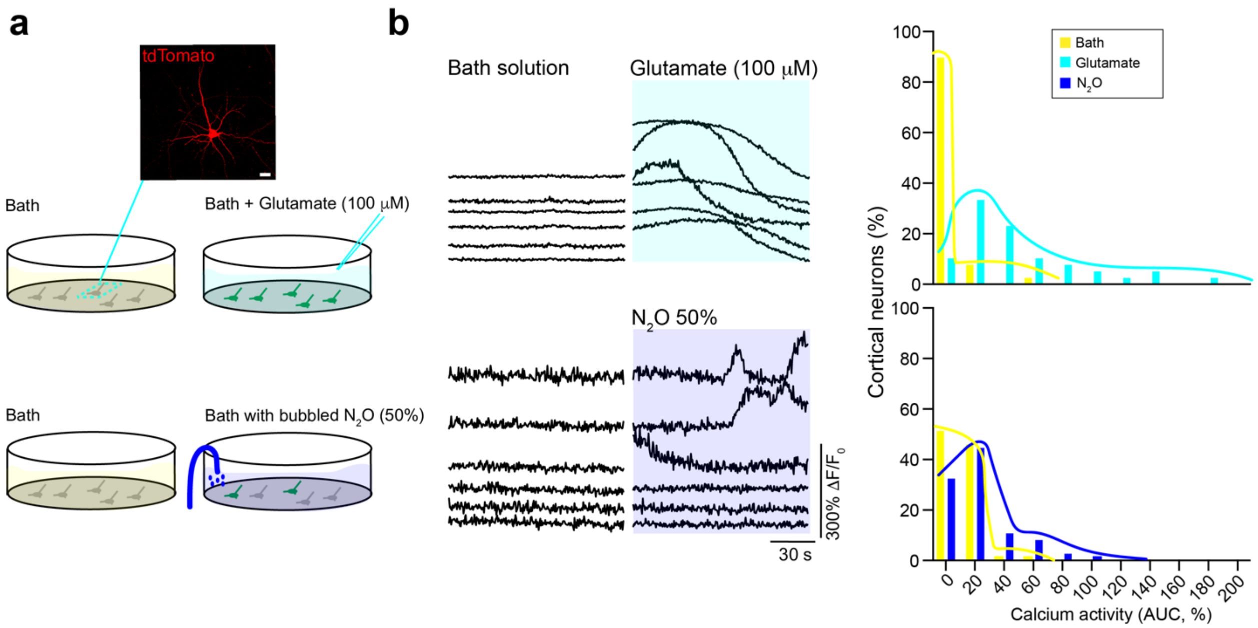

Supplemental Fig. 10. N_2O-induced spontaneous activation of a subset of cortical neurons in vitro.
a, Schematic of low-density cultured cortical neurons plated on PDL coated glass coverslip recorded under baseline conditions in bath (yellow) followed by either glutamate application (teal) or bubbled N_2O in bath (blue). Top, two-photon image showing sparse platting of pyramidal cell (8 DIV) expressing tdTomato under control of CaMKII promoter. Scale bar, 20 µm.
b, GCaMP6 traces (left) and summary of calcium responses (right) from cortical neurons in bath followed by glutamate application (100 uM) or N_2O. In low-density plating conditions neurons are not spontaneously active (left traces), however glutamate application (top; teal shaded region) induces widespread activation of the entire recorded population (Wilcoxon matched-pairs signed rank test, $n = 39$ from 2 coverslips, $P = 1.8 \times 10^{-8}$). On the other hand, N_2O (bottom; blue shaded region) spontaneously activates a subset of cortical neurons from baseline (Wilcoxon matched-pairs signed rank test, $n = 74$ from 3 coverslips, $P = 2.4 \times 10^{-6}$).

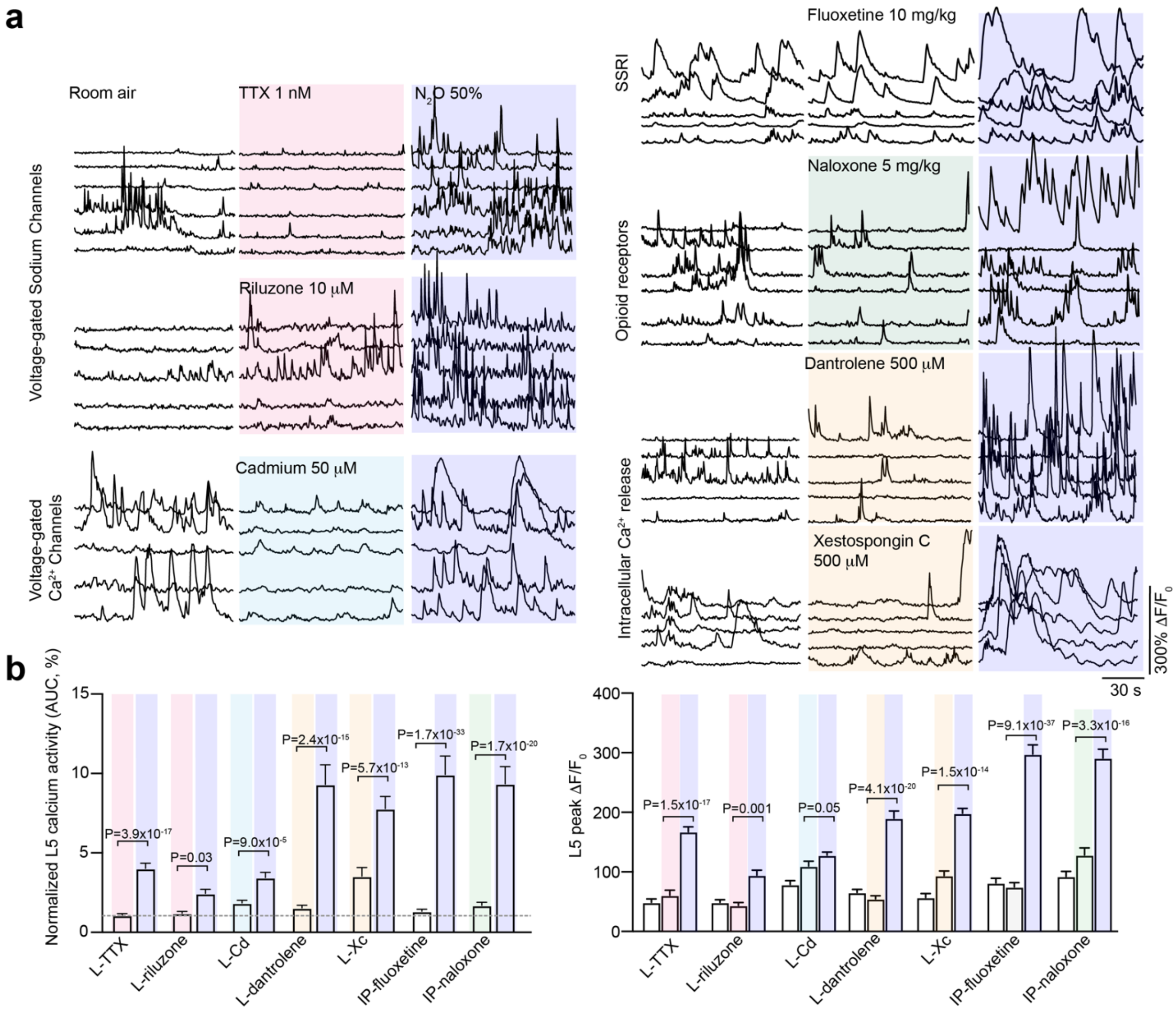

Supplemental Fig. 11. Local pharmacological induced functional attenuation of various channels and receptors prior to N_2O.

a, Representative GCaMP6 traces from individual L5 neurons under room air, either local (L-) or systemic drug administration, followed by N_2O exposure. All drugs were given local except for fluoxetine and naloxone (i.p.). Drug and concentration are noted at middle of each series of traces.

b, Summary of the L5 calcium signals in response to drug manipulation and N_2O. Top, normalized L5 calcium response (Kruskal–Wallis (504): P = 1.3 x 10^{-99} followed by Dunn's multiple comparisons with exact P values shown; L-TTX, n = 91 from 2 mice; L-riluzone, n = 59 from 2 mice; L-Cadmium, n = 109 from 2 mice; L-dantrolene, n = 116 from 3 mice; L-Xc, n = 122 from 3 mice; systemic fluoxetine, n = 144 from 3 mice; systemic naloxone, n = 142 from 3 mice). Bottom, L5 peak $\Delta F/F_0$ (Kruskal–Wallis (818): P = 2.2 x 10^{-160} followed by Dunn's multiple comparisons with exact P values shown). Voltage sodium and calcium channel blockers reduced N_2O-induced L5 activation but could not block it.

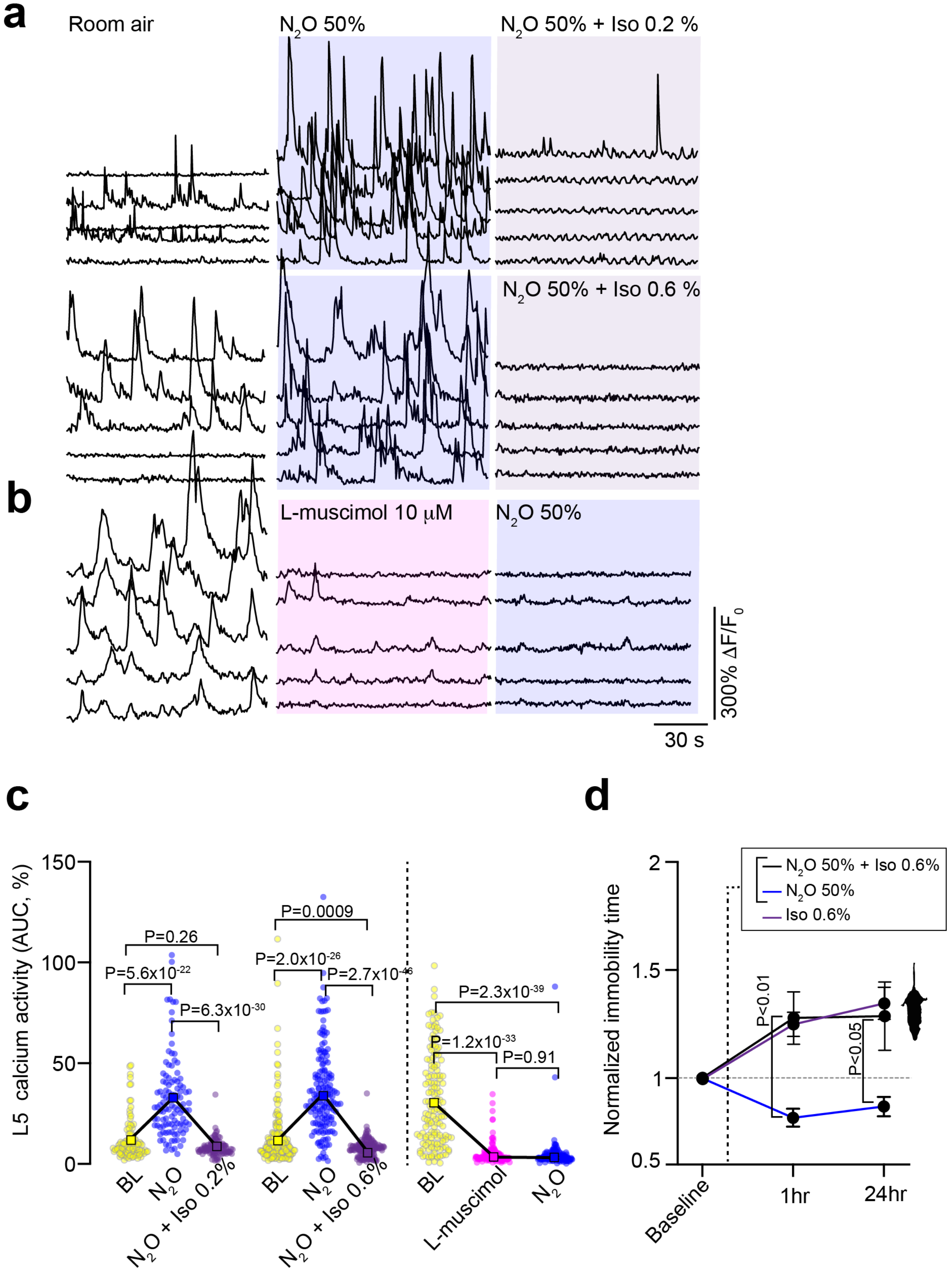

Supplemental Fig. 12. N_2O-induced L5 activity and antidepressant-like effect is occludes by coadministration of GABAergic agents.

a, Representative GCaMP6 traces of individual L5 neuronal responses under room air, N_2O (50%), followed by the N_2O (50%) mixed with isoflurane (Iso; 0.2 or 0.6%).
b, Representative GCaMP6 traces of individual L5 neuronal responses under room air, local application of muscimol (10 uM), followed by the N_2O (50%).
c, N_2O-induced L5 responses are nearly eliminated with both co-inhalation of isoflurane at either concentration (N_2O/Iso 0.2%: *n* = 108 from 3 mice, Kruskal-Wallis (153): $P = 6.4 \times 10^{-34}$ followed by Dunn's multiple comparisons, $P = 6.3 \times 10^{-30}$; N2O/Iso 0.6%: *n* = 160 from 4 mice, Kruskal-Wallis (223): $P = 3.5 \times 10^{-49}$ followed by Dunn's multiple comparisons, $P = 2.7 \times 10^{-46}$) or local muscimol (*n* = 146 from 4 mice, Kruskal-Wallis (216): $P = 1.3 \times 10^{-47}$ followed by Dunn's multiple comparisons, $P = 0.91$)
d, TST immobility time of CORT-treated mice exposed to N_2O 50%, Iso 0.6%, or N_2O 50%/Iso 0.6%. Immobility time of N_2O treated mice (*n* = 7) were significantly different from Iso (*n* = 5) or N_2O/Iso (*n* = 14) at 1 hr (two-way ANOVA: time x treatment $F_{(4, 46)} = 2.6$, $P = 0.002$ post hoc Sidak's test: N_2O vs. Iso $P = 0.0005$, N_2O vs. N_2O/Iso $P = 0.005$) and 24 hr (N_2O vs. Iso $P = 0.002$, N_2O vs. N_2O/Iso $P = 0.04$).

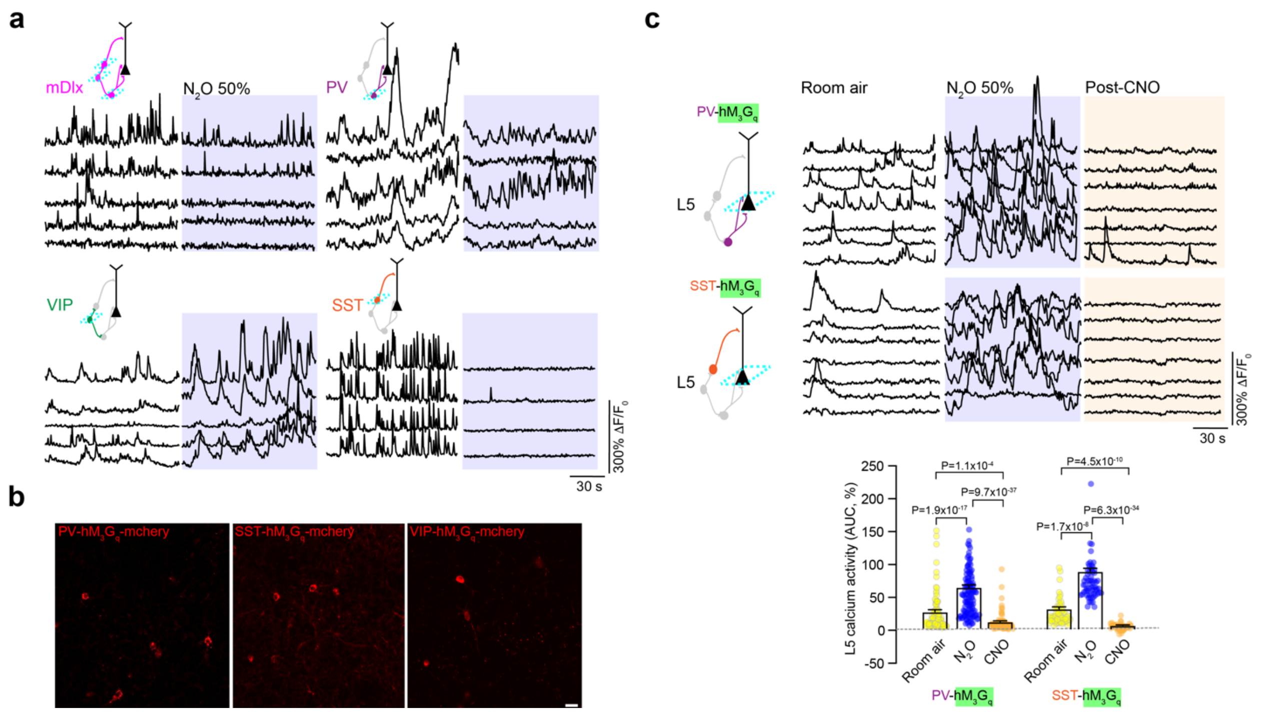

Supplemental Fig. 13. Chemogenetic activation of PV and SST interneurons acutely suppresses N_2O-induced L5 activity.

a, Representative GCaMP6 traces of genetically defined GABAergic interneurons under room air conditions and N_2O exposure.

b, Two-photon images of PV-, SST-, VIP-expressing interneurons expressing DREADD hM_3G_q-mcherry. Cells imaged in layer 2/3 of Cg1. Scale bar, 20 µm.

c, Left, representative GCaMP6 traces of L5 neurons recorded under room air, N_2O, following CNO injection in interneuron-specific Cre-lines (top – PV, bottom – SST) expressing hM_3G_q in Cg1. N_2O-induced L5 activity is acutely silenced by the activation of PV- or SST-expressing interneurons. Right, summary of all L5 cells recorded. N_2O-induced L5 activity does not persist following activation of either PV (Kruskal-Wallis (169): $P = 1.7 \times 10^{-37}$) or SST (Kruskal-Wallis (149): $P = 3.0 \times 10^{-33}$) neurons. Kruskal–Wallis (one-sided test) followed by Dunn's multiple comparisons shown in panels.

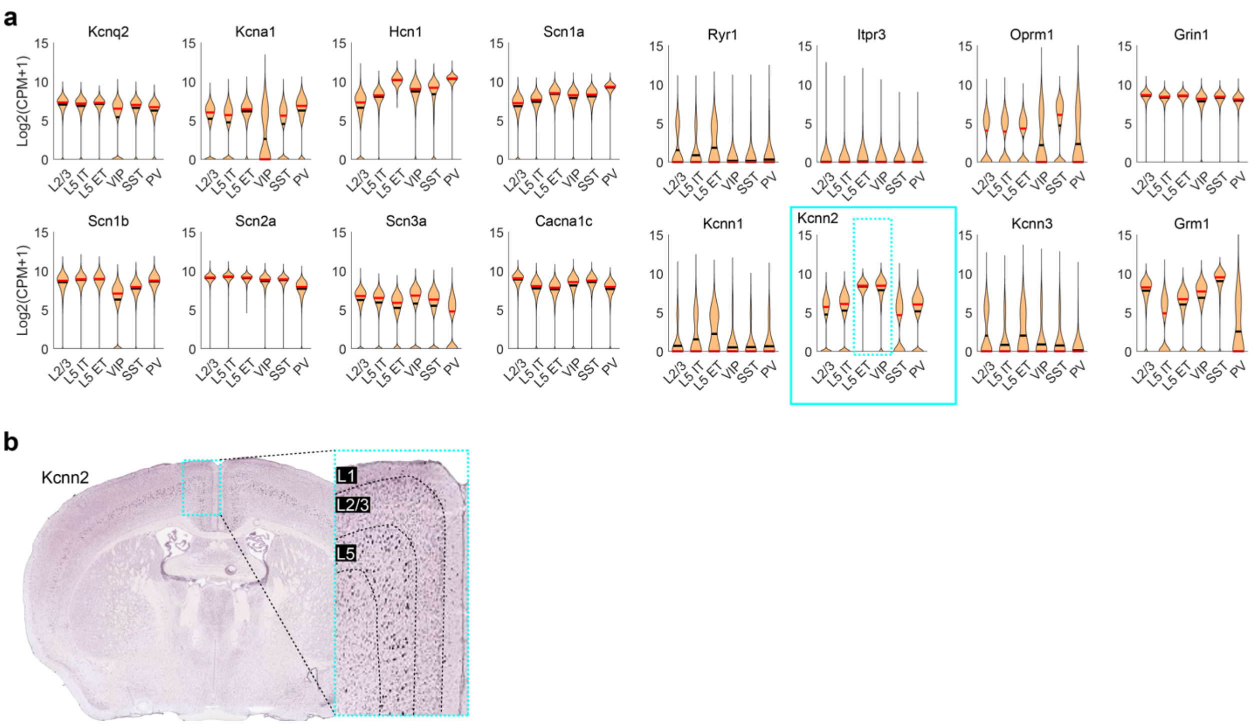

Supplemental Fig. 14. Endogenous SK2 expression in rodent brain is predominantly in layer 5 pyramidal neurons and VIP interneurons.

a, Gene expression levels of candidate channels and receptors (Kcnq2, Kcna1, Hcn1, Scn1a, Scn1b, Scn2a, Scn3a, Cacna1c, Ryr1, Itpr3, Oprm1, Grin1, Kcnn1, Kcnn2, Kcnn3, Grm1) thought to control excitability and calcium dynamics in neuronal cortical cell types (L2/3, L5 IT, L5 ET, VIP, SST, PV; open source whole brain dataset from Allan Brain Cell Atlas, 10x scRNAseq). Distributions of gene expression are shown as violin plots of log2(CPM +1), where black line denotes means, red line denotes medians. CPM = counts per million (transcript reads). Using this screen, we identified the SK2 channel (boxed in teal), encoded by the Kcnn2 gene, to be specifically enriched in Layer 5 ET (extratelencephalic) and VIP neurons (dashed green box) as compared to other cell types (L2/3, L5 IT, SST, PV). Some of these candidates were also empirically confirmed to not block N_2O-induced L5 activity (Supplemental Fig. 11).

b, In situ hybridization of SK2 from Allen Brain mouse atlas shows strong SK2 RNA expression in layer 5 of cortex with sparse labelling in superficial layers. Cortical layers shown in boxed inset.

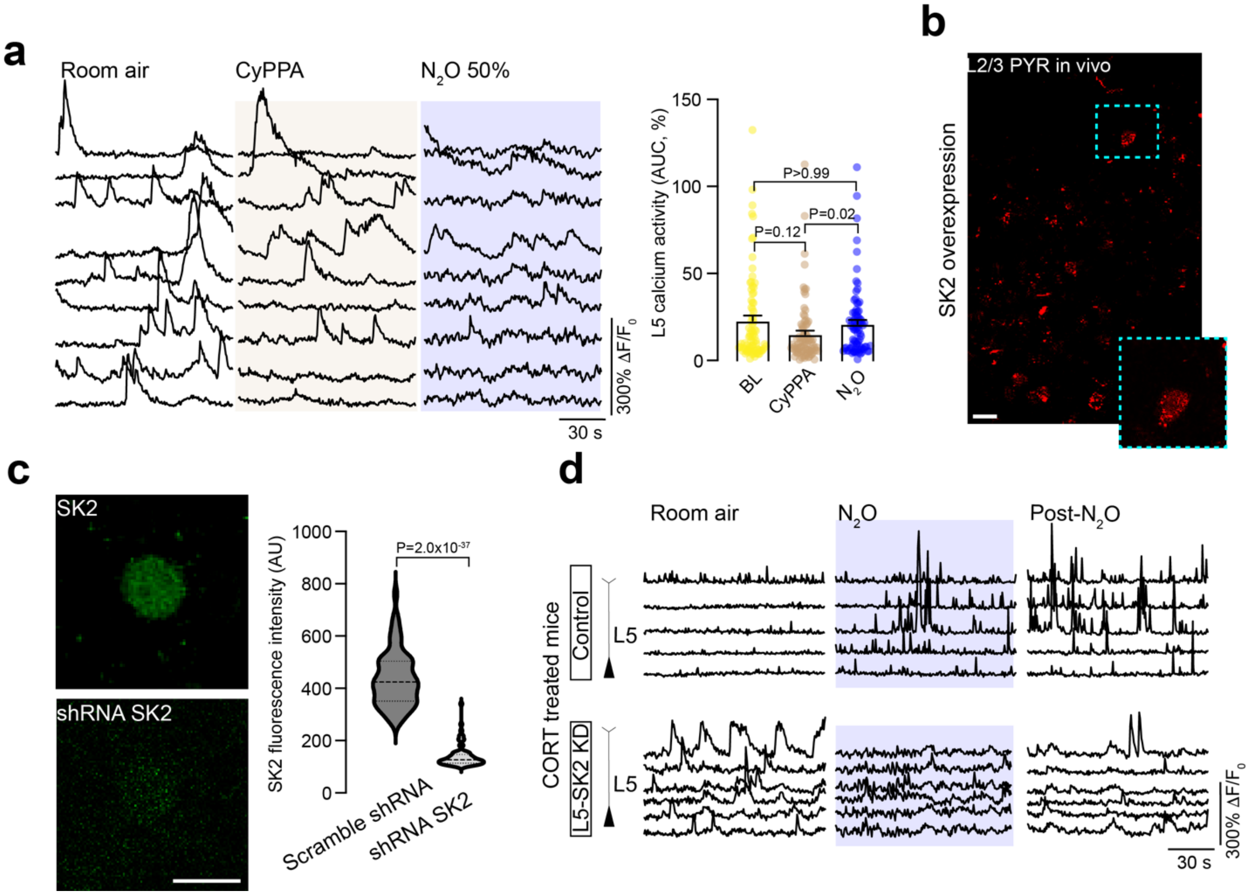

Supplemental Fig. 15. SK2 channel modulation and its impact on N_2O-induced L5 neuronal activity.

a, Representative GCaMP6 traces of individual L5 neurons (left) and all cells avg. calcium response (right; *n* = 80 cells from 2 mice) recorded under wakefulness, local application of SK2 specific activator, CyPPA, (100 µM, 1 µL), and N_2O exposure. SK2 activation by CyPPA blocked N_2O-induced activation of L5 from baseline wakefulness (Kruskal-Wallis (8): $P = 0.02$, followed Dunn's comparison between BL and N_2O: $P > 0.99$).

b, Two-photon image of L2/3 neurons overexpressing SK2-mcherry. Scale bar, 20 µm.

c, Two-photon images (left) and fluorescence intensity quantification (right) of cultured cortical neurons immunostained against SK2 under control/scramble shRNA (*n* = 62 cells) and shRNA-targeted SK2 knockdown (*n* = 73 cells) conditions. SK2-shRNA significantly reduced SK2 expression signals (Mann Whitney rank sum, $P = 2.0 \times 10^{-37}$). Scale bar, 20 µm.

d, Representative GCaMP6 traces of individual L5 responses from CORT-treated animals under wakefulness, N_2O, following N_2O exposure with normal endogenous SK2 expression (top) and under SK2 knockdown condition (bottom).

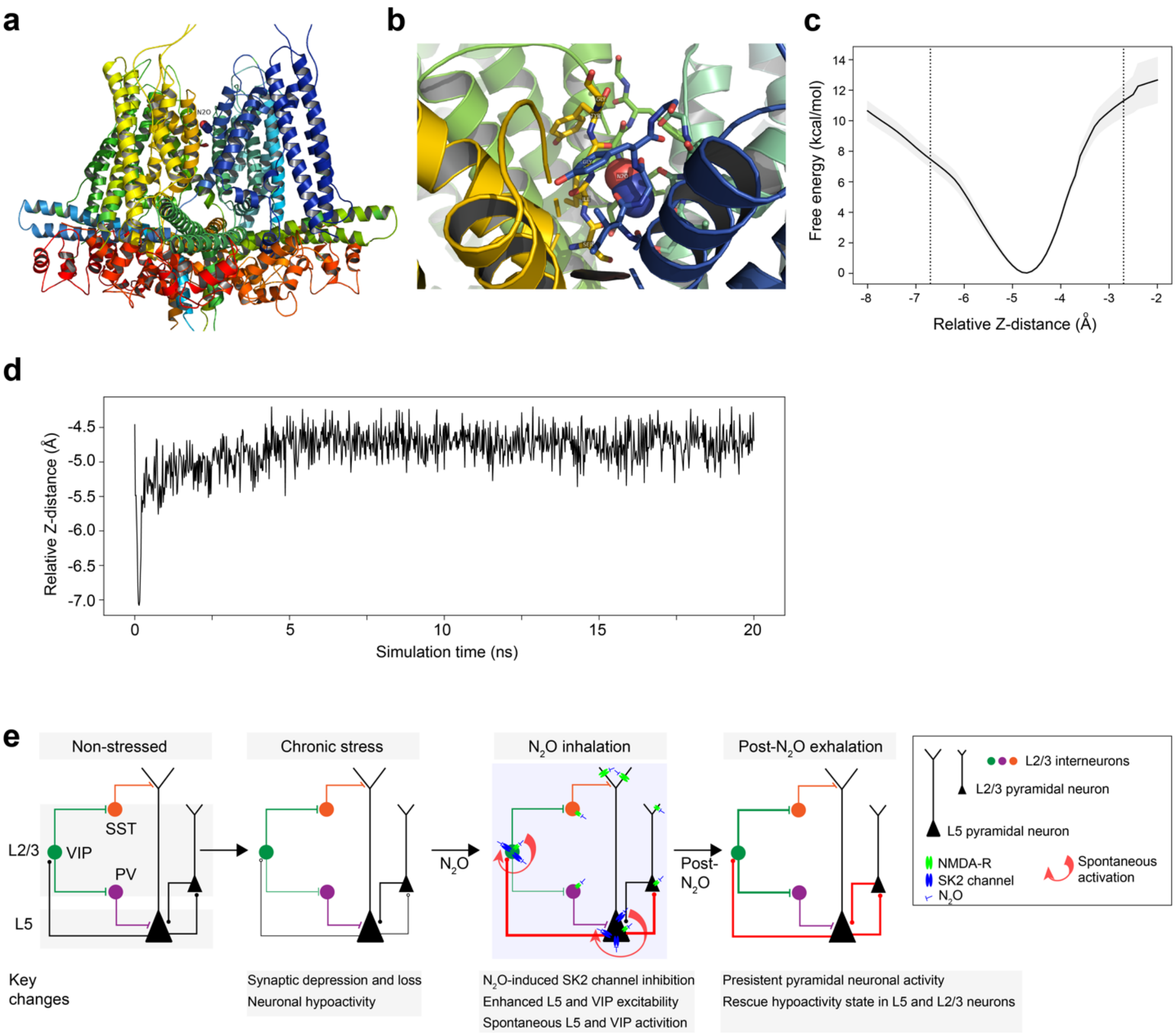

Supplemental Fig. 16. N_2O could act as a channel pore blocker to induce SK2 channel inhibition.
a, Representative snapshot from equilibrium MD simulation of N_2O embedded in selectivity filter of SK2 homology model.
b, Oblique view from extracellular side.
c, PMF curve generated by ABF simulations. Free energy (y-axis) is plotted as a function of relative N_2O z-distance (x-axis). Dotted vertical lines represent displacement of N_2O along pore axis 2 Å in either direction, approaching the top and bottom of the filter. Shaded areas represent standard error with respect to the mean (standard deviation divided by square root of number of replicates).
d, Position of N_2O molecule, relative to C-alpha atoms of Tyr361, z-axis component only, as a function of equilibrium MD simulation time. Note the pore axis is parallel to the z-axis.
e, Working model of N_2O-induced modulation of L5 neurons via SK2 inhibition to drive Cg1 circuit activation to rescue chronic stress-associated hypoactivity state. Chronic stress induces L5 synaptic loss (open circles) and hypoactivity (thin connections) compared to non-stress condition. N_2O-induced SK2 inhibition in cells with high SK2 expression (L5 and VIP cells) drives their rapid spontaneous activation (circular red arrow), which can arise in the presence of NMDA-receptor blockade. L5 neurons recruited during N_2O exposure can engage other cortical circuit elements, *i.e*. superficial L2/3 neurons upon N_2O elimination. Following N_2O treatment, persistent spontaneous activity in both L5 and L2/3 is likely to lead to durable changes circuit function.
