# Supplementary material for: Nitrous oxide activates layer 5 prefrontal neurons via SK2 channel inhibition for antidepressant effect
Source: Nat Commun. 2025 Apr 3;16:2999. doi: 10.1038/s41467-025-57951-y (PMC11968965; doi:10.1038/s41467-025-57951-y)
Supplement: Supplementary file 2 — Description of Additional Supplementary Files [file 41467_2025_57951_MOESM2_ESM.pdf]

Description of Additional Supplementary Files

File name: Supplementary Movie 1
Description: Time lapse movie of nitrous oxide induced layer 5 neuronal activity. Two-photon calcium imaging of layer 5 neurons under room air/wakefulness followed by nitrous oxide (50%) exposure.
