# Supplementary material for: Nitrous oxide activates layer 5 prefrontal neurons via SK2 channel inhibition for antidepressant effect
Source: Nat Commun. 2025 Apr 3;16:2999. doi: 10.1038/s41467-025-57951-y (PMC11968965; doi:10.1038/s41467-025-57951-y)
Supplement: Supplementary file 4 — Reporting Summary [file 41467_2025_57951_MOESM4_ESM.pdf]

nature portfolio

Corresponding author(s): Joe Cichon

Last updated by author(s): Feb. 6, 2024

Reporting Summary

Nature Portfolio wishes to improve the reproducibility of the work that we publish. This form provides structure for consistency and transparency in reporting. For further information on Nature Portfolio policies, see our Editorial Policies and the Editorial Policy Checklist.

Statistics

For all statistical analyses, confirm that the following items are present in the figure legend, table legend, main text, or Methods section.

n/a	Confirmed	
☐	☒	The exact sample size (*n*) for each experimental group/condition, given as a discrete number and unit of measurement
☐	☒	A statement on whether measurements were taken from distinct samples or whether the same sample was measured repeatedly
☐	☒	The statistical test(s) used AND whether they are one- or two-sided<br>*Only common tests should be described solely by name; describe more complex techniques in the Methods section.*
☒	☐	A description of all covariates tested
☐	☒	A description of any assumptions or corrections, such as tests of normality and adjustment for multiple comparisons
☐	☒	A full description of the statistical parameters including central tendency (e.g. means) or other basic estimates (e.g. regression coefficient) AND variation (e.g. standard deviation) or associated estimates of uncertainty (e.g. confidence intervals)
☐	☒	For null hypothesis testing, the test statistic (e.g. *F*, *t*, *r*) with confidence intervals, effect sizes, degrees of freedom and *P* value noted<br>*Give P values as exact values whenever suitable.*
☒	☐	For Bayesian analysis, information on the choice of priors and Markov chain Monte Carlo settings
☒	☐	For hierarchical and complex designs, identification of the appropriate level for tests and full reporting of outcomes
☒	☐	Estimates of effect sizes (e.g. Cohen's *d*, Pearson's *r*), indicating how they were calculated

Our web collection on statistics for biologists contains articles on many of the points above.

Software and code

Policy information about availability of computer code

Data collection	Two-photon images were collected on a Olympus RS DIY two-photon system (tuned to 920 nm) equipped with a Ti:Sapphire laser (Discovery, Coherent). Image acquisition was performed using FV10-ASW v.2.0 software and analyzed post hoc using NIH ImageJ software v.2.1.0. Tail suspension, elevated plus maze, and open field were recorded with ANYMaze software. EEG signals were recorded using a 32-channel headstage (Intan Technologies). Whole-cell recordings were performed using glass pipettes in current-clamp mode.
Data analysis	ImageJ software v.2.1.0. for general image processing, Turbo Reg plugin in ImageJ v.2.1.0 for motion correction, Graph Pad Software v.9.3.1. for calcium analysis and behavioral testing. EEG analysis and mRNA expression plots were performed in MATLAB (2023a) with custom scripts.

For manuscripts utilizing custom algorithms or software that are central to the research but not yet described in published literature, software must be made available to editors and reviewers. We strongly encourage code deposition in a community repository (e.g. GitHub). See the Nature Portfolio guidelines for submitting code & software for further information.

Data

Policy information about availability of data

All manuscripts must include a data availability statement. This statement should provide the following information, where applicable:

- Accession codes, unique identifiers, or web links for publicly available datasets
- A description of any restrictions on data availability
- For clinical datasets or third party data, please ensure that the statement adheres to our policy

Figures and associated data will be available to the scientific community via FigShare.

Research involving human participants, their data, or biological material

Policy information about studies with human participants or human data. See also policy information about sex, gender (identity/presentation), and sexual orientation and race, ethnicity and racism.

Reporting on sex and gender	N/A
Reporting on race, ethnicity, or other socially relevant groupings	N/A
Population characteristics	N/A
Recruitment	N/A
Ethics oversight	N/A

Note that full information on the approval of the study protocol must also be provided in the manuscript.

Field-specific reporting

Please select the one below that is the best fit for your research. If you are not sure, read the appropriate sections before making your selection.

☒ Life sciences ☐ Behavioural & social sciences ☐ Ecological, evolutionary & environmental sciences

For a reference copy of the document with all sections, see nature.com/documents/nr-reporting-summary-flat.pdf

Life sciences study design

All studies must disclose on these points even when the disclosure is negative.

Sample size	No statistical tests were used to predetermine sample sizes, but sample sizes for this study are similar to those generally employed in the field (e.g., PMID: 25822789, 28092659, 31972184, 28671692, 24904169, 30792151, 29731710).
Data exclusions	No animals were excluded from analysis. Imaging with excessive movement were removed from analysis. Cells with GCaMP localized to nucleus were excluded from analysis.
Replication	Experiments were replicated in at least 3 separate mice.
Randomization	Animals were assigned to nitrous oxide or oxygen randomly.
Blinding	J.C. was not blind to treatment or genotype during imaging or behavior experiments because J.C carried out all experiments except for Figure 6. In Figure 6, X.L., C. F. Z, and S.M. were not blinded to treatments for electrophysiological recordings.

Reporting for specific materials, systems and methods

We require information from authors about some types of materials, experimental systems and methods used in many studies. Here, indicate whether each material, system or method listed is relevant to your study. If you are not sure if a list item applies to your research, read the appropriate section before selecting a response.

Materials & experimental systems

n/a	Involved in the study
☐	☒ Antibodies
☒	☐ Eukaryotic cell lines
☒	☐ Palaeontology and archaeology
☐	☒ Animals and other organisms
☒	☐ Clinical data
☒	☐ Dual use research of concern
☒	☐ Plants

Methods

n/a	Involved in the study
☒	☐ ChIP-seq
☒	☐ Flow cytometry
☒	☐ MRI-based neuroimaging

Antibodies

Antibodies used	SK2 was immunostained with rabbit monoclonal antibody (1:200; Boster Biological Technology, A05055). NR1 was identified with a NMDA-receptor 1 mouse monoclonal antibody (1:100; Fisher Scientific, 30-050-0). Secondary antibodies including anti-mouse Cy3 or anti-rabbit Cy3 (Jackson ImmunoResearch) were used.
Validation	SK2 channel overexpression in cells that normally lack endogenous SK2 expression was accomplished with expression of Cre-dependent mKCNN2 under the human synapsin-1 promoter in Cre-expressing cells or Cre-positive mice (AAV9-hSyn1-DIO-mKCNN2-2A-mCherry; Vector Biolabs; reference sequence: NM_001312905; titer: 8.5x 1012 GC/mL)(Fig. 7c-d). SK2 channel knockdown in vivo and in vitro was accomplished by expression of Cre-dependent mKCNN2-shRNAmir under the CAG promoter in Cre-expressing cells or Cre-positive mice (AAV9-CAG-DIO-GFP-mKCNN2-shRNAmir#1; Vector Biolabs; reference sequence: NM_080465; titer: 6.4x 1012 GC/mL) (Fig. 7c-e, Supplemental Fig. 15). Based on shRNAmir#1 (5'- GCT GTCCATGTGAACGTATAATTCCGTTTTGG CCAC TGACTGACGGAATTATGTTCACATGGA CAG-3') screening, shRNAmir#1 yields 83% knockdown of mRNA (communication from Vector Biolabs). We confirmed this knockdown efficacy in cultured cortical neurons by measuring SK2 immunofluorescence in cells with or without shRNAmir#1 expression (~68% reduction of SK2 immunofluorescence in GFP-mKCNN2-shRNAmir#1 expressing cells as compared to GFP-scramble shRNA).<br><br>NMDA-receptor NR1 subunit knockdown in vivo and in vitro was accomplished by packaging GRIN1 siRNA oligos in AAV9 (Abmgood; Cat. No. 22671174; reference sequence: NM_008169) (Fig. 3c, e, Supplemental Fig. 8). A scrambled siRNA AAV was used in parallel for control (Abmgood, 01509). Based on target sequences (Target a - 7 ACCATGCACCTGCTGACATTCGCCCTGCT, Target b - 969 GGTGCTGATGTCTTCCAAGTATGCAGATG, Target c - 1507 GGAGAGCTGCTCAGTGGTCAAGCAGACAT, Target d - 2591 ATAGAAAGAGTGGTAGAGCAGAGCCCGAC) siRNA oligos expression should produce a 70% knockdown of mRNA (communication from Abmgood). We confirmed this knockdown efficacy in cultured cortical neurons by measuring NR1 immunofluorescence in cells with siRNA to GRIN1 as compared to scrambled siRNA control (~50% reduction of NR1 expression) (Supplemental Fig. 8).

Animals and other research organisms

Policy information about studies involving animals; ARRIVE guidelines recommended for reporting animal research, and Sex and Gender in Research

Laboratory animals	The study was approved by the University of Pennsylvania Animal Care and Use Committee (approved protocol no. 807237). All animals were treated in strict accordance with National Institutes of Health (NIH) and institutional guidelines, the United States Public Health Service Policy on Humane Care and Use of Laboratory Animals, and the Guide for the Care and Use of Laboratory Animals. All mice were maintained at the University of Pennsylvania Perelman School of Medicine John Morgan animal facility with controlled temperature and humidity conditions and had free access to food and water/CORT water.<br><br>In Fig. 1 and 5, GCaMP6f expression was driven using AAV in Rbp4-Cre (GENSAT project at Rockefeller University, 031125), Tlx3-Cre (GENSAT; PL56), Colgalt2-Cre (GENSAT; NF107), PV-IRES-Cre (Jackson Laboratory, 008069), Sst-IRES-Cre (Jackson Laboratory, 013044) and VIP-IRES-Cre (Jackson Laboratory, 010908) mice. Imaging was performed in 1 to 2-month-old mice, using both sexes, after at least 4weeks of AAV expression. Mice were group-housed in temperature and humidity-controlled rooms on a 12-hour light/dark cycle after injections.
Wild animals	No wild animals were used in this study.
Reporting on sex	Both males and females were used for behavior and imaging experiments but too few for statistical analysis of sex effect.
Field-collected samples	No field collected sampled were used in this study.
Ethics oversight	Institutional Animal Care and Use Committee, University of Pennsylvania.

Note that full information on the approval of the study protocol must also be provided in the manuscript.

Plants

Seed stocks	N/A
Novel plant genotypes	N/A
Authentication	N/A
